# Comparison of CAD/CAM manufactured implant-supported crowns with different analyses

**DOI:** 10.1186/s40729-020-00267-x

**Published:** 2020-10-27

**Authors:** Elif Yeğin, Mustafa Hayati Atala

**Affiliations:** 1 Department of Prosthodontics, Faculty of Dentistry, Biruni University, Istanbul, Turkey; 2 Department of Prosthodontics, Faculty of Dentistry, Medeniyet University, Istanbul, Turkey

**Keywords:** CAD/CAM, Ceramics, Dental implant, Finite element analysis, Load-to-failure test

## Abstract

**Background:**

Present study compared the failure load of CAD/CAM-manufactured implant-supported crowns and the stress distribution on the prosthesis-implant-bone complex with different restoration techniques.

**Methods:**

The materials were divided into four groups: group L-M: lithium disilicate ceramic (LDS, monolithic), group L-V: LDS ceramic (veneering), group ZL-M: zirconia-reinforced lithium silicate ceramic (ZLS, monolithic), group ZL-V: ZLS ceramic (veneering). Crown restorations were subjected to load-to-failure test (0.5 mm/min). Failure loads of each group were statistically analyzed (two-way ANOVA, post hoc Tukey HSD, *α* = 0.05). Finite element analysis (FEA) was used to compare the stress distribution of crown restorations.

**Results:**

Group L-M had the highest failure load (2891.88 ± 410.12 N) with a significant difference from other groups (*p* < 0.05). Although there was a significant difference between group ZL-M (1750.28 ± 314.96 N) and ZL-V (2202.55 ± 503.14 N), there was no significant difference from group L-V in both groups (2077.37 ± 356.59 N) (*p* > 0.05).

**Conclusions:**

The veneer application had opposite effects on ceramics, increased the failure load of ZLS and reduced it for LDS without a statistically significant difference. Both materials are suitable for implant-supported crowns. Different restorative materials did not influence the stress distribution, but monolithic restorations reduced the stress concentration on the implant and bone.

## Background

Implants have been successfully used to replace missing teeth for many years. Notwithstanding the high success rates, complications such as screw loosening and/or fracture, prosthesis fracture, and even implant fracture are inevitable [1, 2]. The reasons of the complication may be related to decreased proprioception and low tactile sensitivity [3] which makes implant-supported crowns more susceptible to occlusal overload than tooth-supported crowns [4].

Metal-based superstructures are frequently used for implant-supported restorations [5]. Since the metal grayish shade and opaque structure prevent light transmission, they demonstrate limited esthetic results that cannot meet esthetic demands [6, 7]. Besides this esthetic disadvantage, the more important problem is the biological one, the potential corrosion process between the titanium and metal-based superstructure, which can cause implant failures [8]. Therefore, natural-looking appearance, biocompatibility, and improved mechanical properties have led to the use of all ceramics in implant-supported restorations, as in fixed partial dentures [9, 10]. In nowadays, continual marketing of new all-ceramic systems and advances in CAD/CAM (computer-aided design/computer-aided manufacturing) technology make the use of all ceramics a more preferable alternative that can be fabricated monolithic or manually veneered [11].

Lithium disilicate (LDS) which is the most popular glass ceramic has small needle-shaped crystals and glass matrix [12]. It can be manufactured by CAD/CAM (IPS e.max CAD, Ivoclar Vivadent) and can be used for substructure or full-contour restorations (monolithic restorations) [13]. Recently, a new glass ceramic, zirconia-reinforced lithium silicate (ZLS; Vita Suprinity, Vita Zahnfabrik, Celtra Duo, Dentsply DeTrey) has been marketed (Table 1) [14].

**Table 1:** The materials used in the study

Material	Chemical composition (%)	Coefficient of thermal expansion (10^**−6**^ K^**−1**^)	Flexural strength (MPa)	Manufacturer
IPS e.max CAD; lithium disilicate glass ceramic (LDS)	SiO_2_ (57–80), Li_2_O (11–19), K_2_O (0–13), P_2_O_5_ (0–11), ZrO_2_ (0–8), ZnO (0–8), Al_2_O_3_ (0–5), MgO (0–5), coloring oxides (0–8)	10.2	360	Ivoclar Vivadent
IPS e.max Ceram; low-fusing nano-fluorapatite glass-ceramic	SiO_2_ (60–65), Al_2_O_3_ (8–12), Na_2_O (6–9), K_2_O (6–8), ZnO (2–3), CaO, P_2_O_5_ ve F (2–6), other oxides (2–8.5), pigments (0.1–1.5)	9.5	90	Ivoclar Vivadent
Vita Suprinity; zirconia-reinforced lithium silicate glass ceramic	ZrO_2_ (8–12), SiO_2_ (56–64), Li_2_O (15–21), La_2_O_3_ (0.1), pigments (< 10), various (> 10)	12.3	420	Vita Zahnfabrik
Vita VM 11; low-fusing feldspar ceramic	SiO_2_ (62–65), Al_2_O_3_ (8.5–12), Na_2_O (5–7.5), K_2_O (9–12), CaO (1–2), ZrO_2_ (< 1), B_2_O_3_ (4–6)	11.6	100	Vita Zahnfabrik

This material has a fine-grained, homogenous microstructure [15] and flexural strength value comparable to lithium disilicate [16]. It is reinforced with approximately 10% zirconia [14] which strengthens the ceramic structure by avoiding crack propagation [17] and demonstrates enhanced mechanical and esthetic features due to the combination of the positive material properties of zirconia and glass ceramic [18]. In a clinical study of Zimmermann et al., it was concluded that ZLS ceramic restorations had a high clinical success rate (96.7%) after 12 months [19].

In the literature, a limited number of studies have demonstrated the outcomes of all ceramic implant-supported restorations. Therefore, the aim of this study was to compare the failure load of CAD/CAM-manufactured implant-supported crowns with different restoration techniques and stress distribution on prosthesis-implant-bone complex. The null hypotheses were that (1) there were no differences in the failure load of two different monolithic glass ceramic crowns; (2) veneering technique did not affect the failure load of the different glass ceramics; and (3) veneered crowns have similar failure load values.

## Methods

### Preparation of test groups

This study tested the current glass ceramic ZLS by comparing LDS with monolithic and conventional veneering techniques in implant-supported crowns: group L-M: lithium disilicate ceramic (monolithic), group L-V: lithium disilicate ceramic (conventional veneering), group ZL-M: zirconia-reinforced lithium silicate ceramic (monolithic), group ZL-V: zirconia-reinforced lithium silicate ceramic (conventional veneering) (Table 2).

**Table 2:** The materials in the groups

Groups	***N***	Materials
L-M	12	IPS e-max CAD IPS e.max CAD glaze
L-V	12	IPS e-max CAD e.max Ceram Dentin IPS e.max Ceram Glaze
ZL-M	12	Vita Suprinity Vita Akzent Plus
ZL-V	12	Vita Suprinity VM-11 Vita Akzent Plus

Bone-level implants with a diameter of 4.1 mm and a length of 12 mm (Hager & Meisinger GmbH, Germany) and prefabricated cement-type abutments (5 mm in diameter, 6 mm in height, serial number A66756, Hager & Meisinger GmbH, Germany) were used for simulating the clinical situation of a missing mandibular first molar teeth.

Forty-eight implants (Hager & Meisinger GmbH, Germany) were embedded in a plastic ring using an auto polymerizing acrylic resin (Vertex-Dental BV, Zeist, Holland) vertically to the horizontal plane. The same number of prefabricated abutments (Hager & Meisinger GmbH, Germany) was fixed to them at 30 N.cm of torque according to the manufacturer’s instructions.

### Manufacture of the crowns

**Fig. 1:**
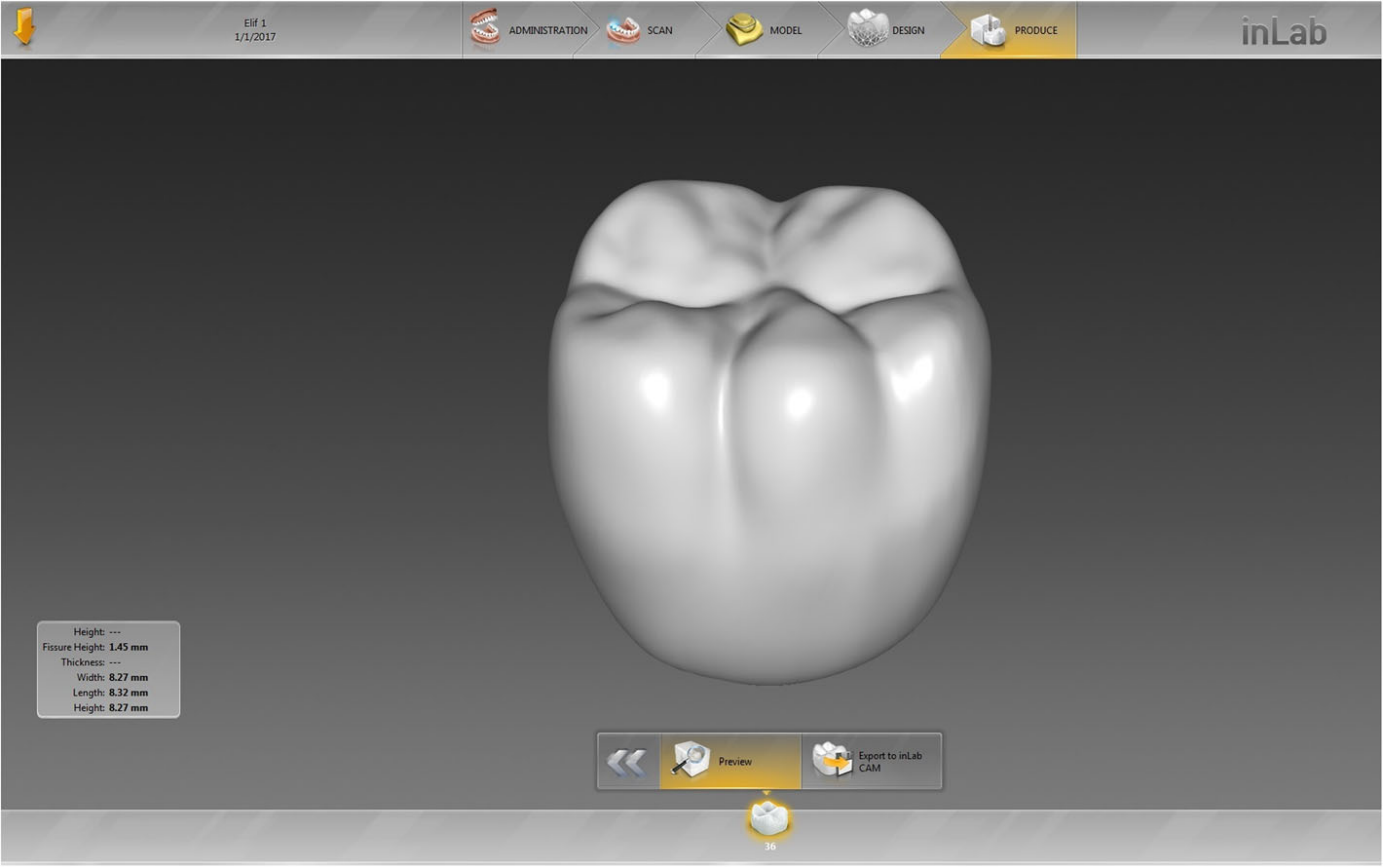
Crown restoration design

Contrast spray (IPS Contrast Spray Labside; Ivoclar Vivadent, Schaan, Liechtenstein) was scattered on an implant-abutment complex for optical impression (InEos Blue, Sirona Dental Systems, GmbH, Bensheim, Germany). After scanning abutment, the crown design was performed with Cerec InLab V15.0 based on the mandibular first molar tooth morphology (Fig. 1) and was calibrated to 1.5 mm in fossa, 2 mm in cusps, and the entire crown. “Multilayer” was selected among different design modes. In this design technique, the fully anatomical shape can be splinted into a core and a covering crown. Therefore, the cores of the two veneering groups and monolithic groups were fabricated in a standardized way.

For monolithic restorations, the design mode “unsplit” was defined to obtain fully anatomical crowns. A total of 24 monolithic crowns (*n* = 12 for group L-M and *n* = 12 for group ZL-M) were fabricated by a milling machine (InLab MC XL, Sirona Dental Systems, GmbH, Bensheim, Germany).

All crowns were subjected to a combination firing that included crystallization and glaze firing according to each manufacturer’s guidelines in the ceramic furnace (Vita Vacumat 6000 M, Vita Zahnfabrik, Bad Sackingen, Germany).

For veneered restorations, the design mode was changed to “split,” and the core was constructed in 0.6-mm thickness. In group L-V (*n* = 12), e.max CAD core and nano-fluorapatite veneering ceramic (e.max Ceram Dentin, Ivoclar Vivadent) and in group ZL-V (*n* = 12), Vita Suprinity core and low-fusing fine-structure feldspar ceramic (VM-11, Vita Zahnfabrik) were used. The restorations of group L-V were conducted to wash firing and two dentin firing according to the manufacturer’s instructions. For wash firing, e.max Ceram Dentin was mixed with IPS Build-Up Liquids allround (Ivoclar Vivadent) and applied to the entire core as a thin coat. All cores were than layered with the same porcelain using a silicon index guide obtained from a monolithic crown to standardize the thickness of the veneering porcelain. Since the wash firing was not recommended in group ZL-V, only two dentin-firing cycles were performed with Vita VM-11 Dentin materials, and the silicon index was used as in group L-V. After the veneering procedure was completed, all restorations were glazed.

All crowns were cemented to abutments with zinc phosphate cement (Adhesor, Spofa-Dental, Czech Republic) with a standard load of 30 N. Cemented crowns were then stored in distilled water at 37 °C for 24 h before load-to-failure testing.

### Load-to-failure testing

The test was carried out with a dynamic/static testing machine (Instron 8801, INSTRON Ltd, England) at a cross-head speed of 0.5 mm/min. The vertical load was applied with a stainless steel ball (5.0 mm in diameter) placed on the occlusal surface of the crowns. The software (Bluehill) of the testing machine recorded the failure load of the crowns.

### Statistical analysis

The statistical analysis was performed with SPSS 24.0 (SPSS Inc, Chicago, USA). The Kolmogorov–Smirnov normality test was used to evaluate whether the data distribution of the groups was normal. The homogeneity of the variances was analyzed by Levene’s test. Since test results indicated that data distribution of the groups was normal and the variances were homogenous, two-way analysis of variance (ANOVA) and Tukey HSD test were used to compare the groups. *p* < 0.05 was determined as statistically significant. According to the power analysis (PASS v.11) with 90% power and 95% confidence intervals of previous studies, at least 10 samples per group were required [5, 11].

### Fractographical analysis

After the load-to-failure test, each sample was evaluated for chipping, delamination, or bulk fracture. And also, all abutments were checked for the presence of screw loosing or fracture.

### Finite element analysis (FEA)

The bone tissue design was obtained by CT scan of a patient in the Materialise Mimics Software (version 17, Leuven, Belgium). The crown design and scan data of the implant and abutment obtained by the CAD/CAM scanner were exported in “.stl” file format. These three-dimensional (3D) data were combined with the bone tissue design in the Hypermesh Software (version 2013, Altair Hyperworks, Troy, MI, USA). All 3D structures were modeled with tetrahedral elements with four nodes. Crown models consist of 126,810 nodes and 697,260 elements. Young’s modulus and Poisson ratio of the materials used in the study are listed in Table 3 with references.

**Table 3:** The properties of the materials used in FEA and the references of these values

Material	Young’s modulus (GPa)	Poisson ratio	Reference
**E.max CAD**	95	0.20	[1]
**Vita Suprinity**	65	0.23	[2]
**Vita VM 11**	65	0.23	*
**E.max Ceram**	64	0.23	[4]
**Implant and abutment**	114	0.34	[5]
**Cortical bone**	13.7	0.3	[5]
**Spongious bone**	1	0.3	[5]

*Young’s modulus provided by the manufacturer. The Poisson ratio is unknown and the value of E.max Ceram was used

Finite element analysis was performed with the Abaqus Software (version 6-14.1). A total of 300 N (an average occlusal force in molar area) static vertical load was applied centrally to three points of (100 N) the occlusal surface of each crown [20, 21] as in the load-to-failure test. Von Mises, Pmax (tensile strength), and Pmin (compressive strength) were calculated, and the stress distributions were determined.

## Results

Descriptive analysis (mean, standard deviation (SD), minimum, maximum) of the groups is presented in Table 4.

**Table 4:** Descriptive statistical analysis of the groups

Group	***N***	Mean (***N***)	Standard deviation	Minimum	Maximum
**L-M**	12	2891.88^a^	410.12	2079.74	3486.96
**L-V**	12	2077.37^bc^	356.59	1220.96	2493.39
**ZL-M**	12	1750.28^c^	314.96	1084.36	2163.95
**ZL-V**	12	2202.55^b^	503.14	1292.20	2912.81

Different superscript letters indicate statistically significant differences (*p* < 0.05)

**Fig. 2:**
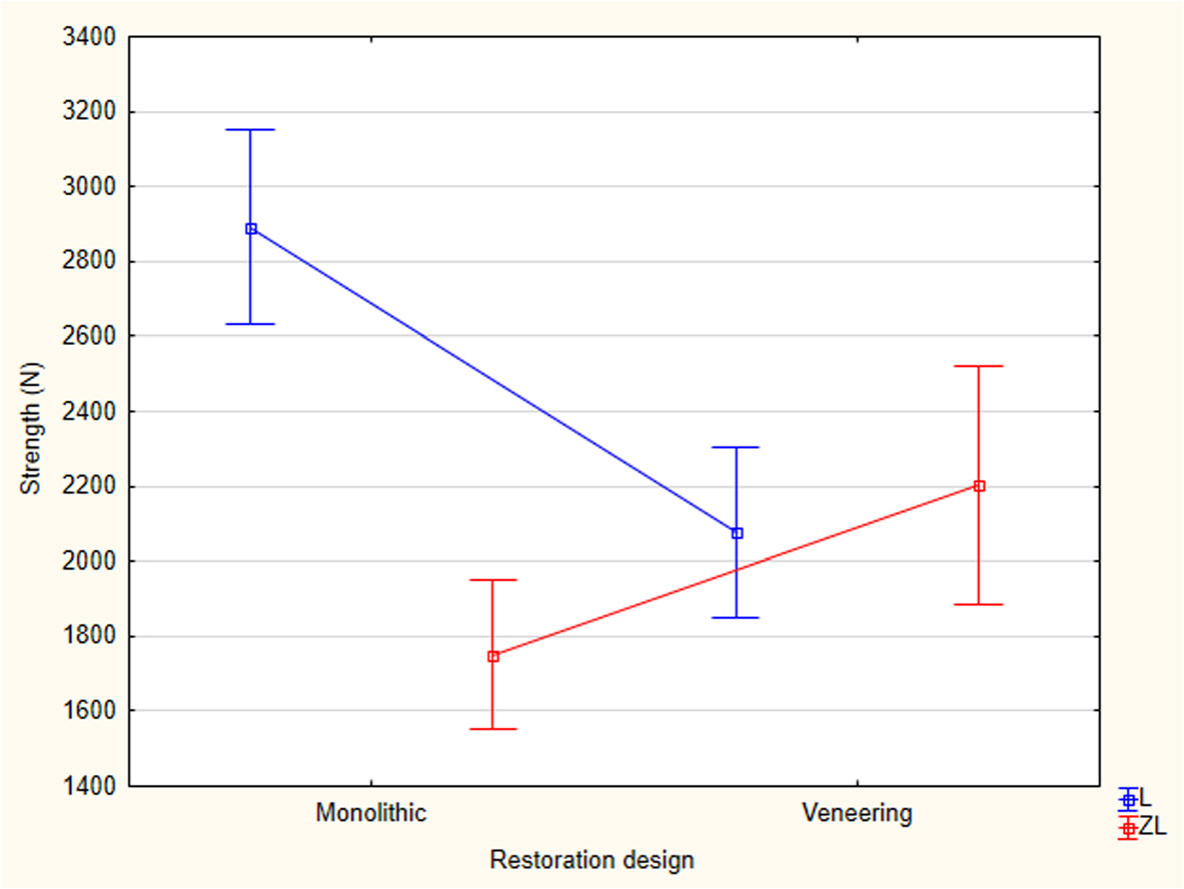
The graph of the interaction of the materials and restoration design

Group L-M exhibited the highest failure load values (2891.88 N ± 410.12 N), and the lowest values were observed in group ZL-M (1750.28 N ± 314.96 N). Two-way ANOVA indicated a statistically significant difference between materials and veneering technique (*p* = 0.00 < 0.05). Tukey’s test confirmed statistically significant differences observed between the groups ZL-V and ZL-M (*p* = 0.04 < 0.05) and L-V and L-M (*p* = 0.00 < 0.05). To compare the restoration design, monolithic design demonstrated a statistically significant difference between materials (*p* = 0.00 < 0.05; L-M > ZL-M). Although the veneering technique did not present a statistically significant difference between the materials (*p* = 0.87 > 0.05), this technique had opposite effects on two different ceramics that increased the failure load of ZLS ceramic and decreased it for LDS ceramic (Fig. 2).

### Fracture analysis

No chipping or delamination was observed after the load-to-failure test since bulk fracture was observed in all groups. The fracture pattern was mostly the central groove direction in the veneered groups and the fissural direction with multiple fragments in the monolithic groups. Screw loosing was found in 5 specimens of groups L-M and ZL-V, 2 specimens of group L-V, and 1 specimen of group ZL-M.

### Finite element analysis

**Fig. 3:**
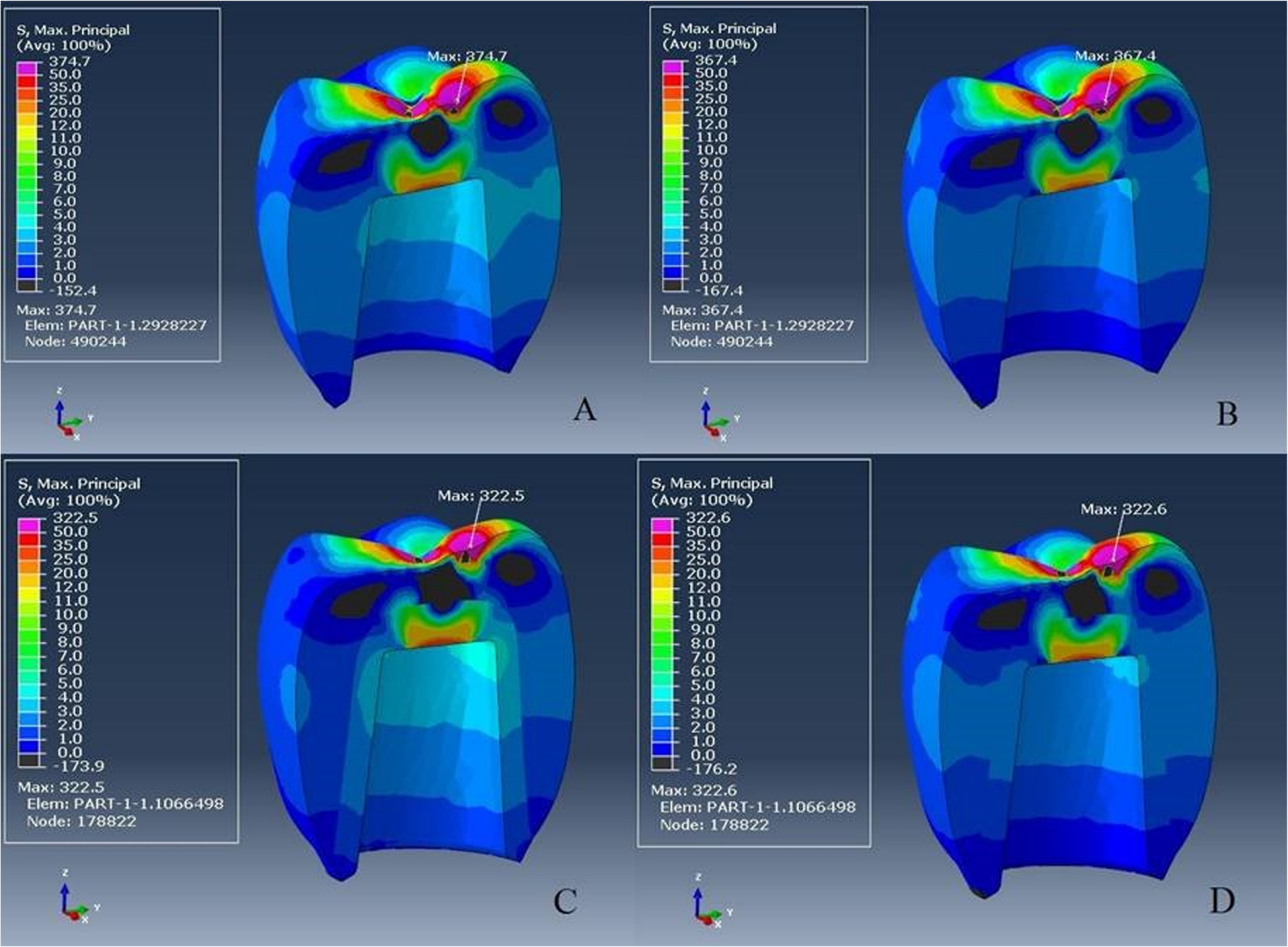
**a**–**d** Maximum principal stress distribution on crown restoration. From **a** to **d**: L-M, ZL-M, L-V, and ZL-V respectively

The Pmax value of crown restoration of group L-M was 374.7 MPa, and the tensile stresses were concentrated in the load application site and the coronal part of the abutment (Fig. 3a). Von Mises stresses were also concentrated at the load application site and transferred to the coronal part of the abutment. The Pmax value of crown restoration of group ZL-M was 367.4 MPa, which was lower than other monolithic restoration (Fig. 3b). Tensile stress distribution and Von Mises stresses were similar to those in group L-M.

The tensile stresses in group L-V and group ZL-V were concentrated at the load application site on the veneering ceramic with relatively similar Pmax values (322.5–322.6 MPa; Fig. 3c, d) and also the occlusal part of the cores (34.7–31.5 MPa).

**Fig. 4:**
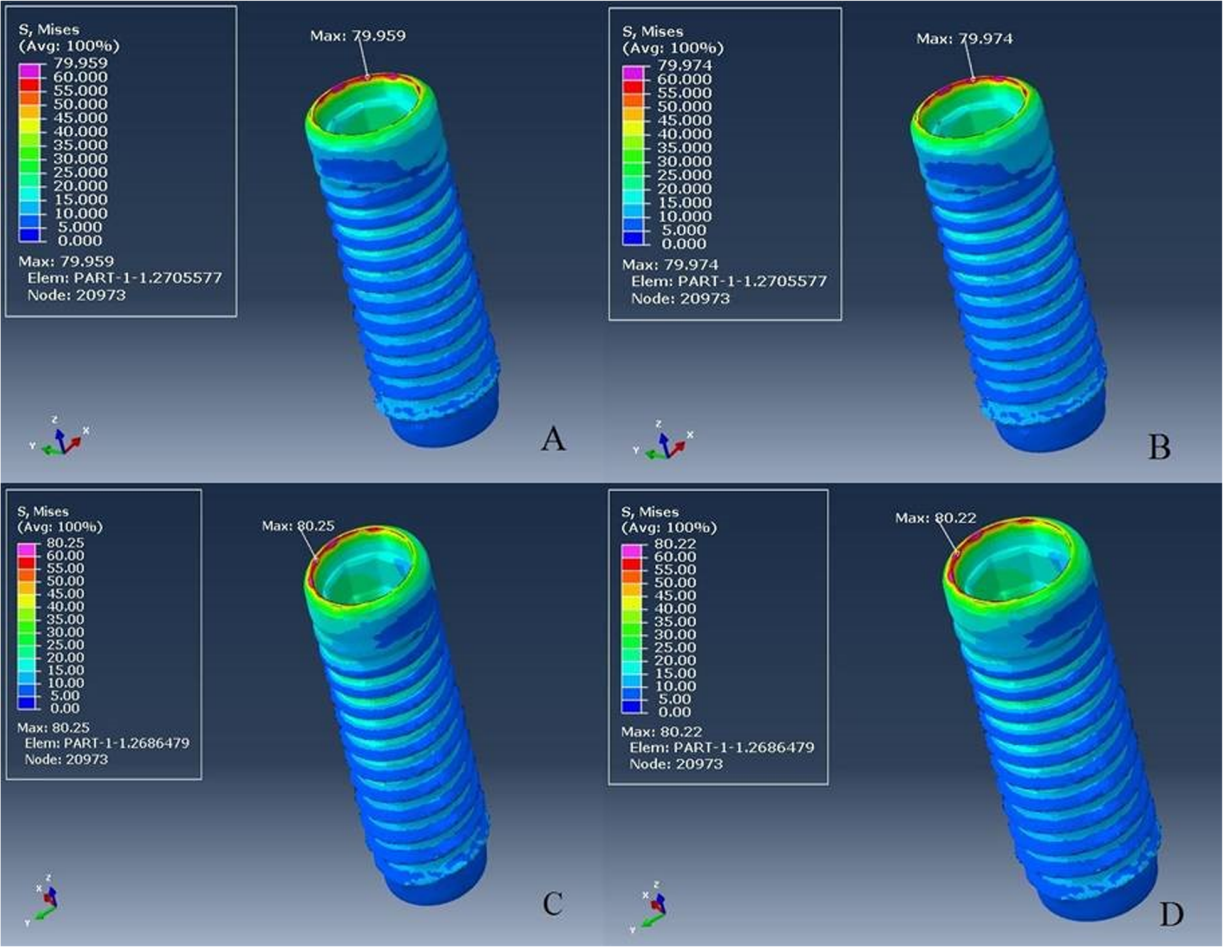
**a**–**d** Von Mises stress distribution on implant. From **a** to **d**: L-M, ZL-M, L-V, and ZL-V respectively

**Fig. 5:**
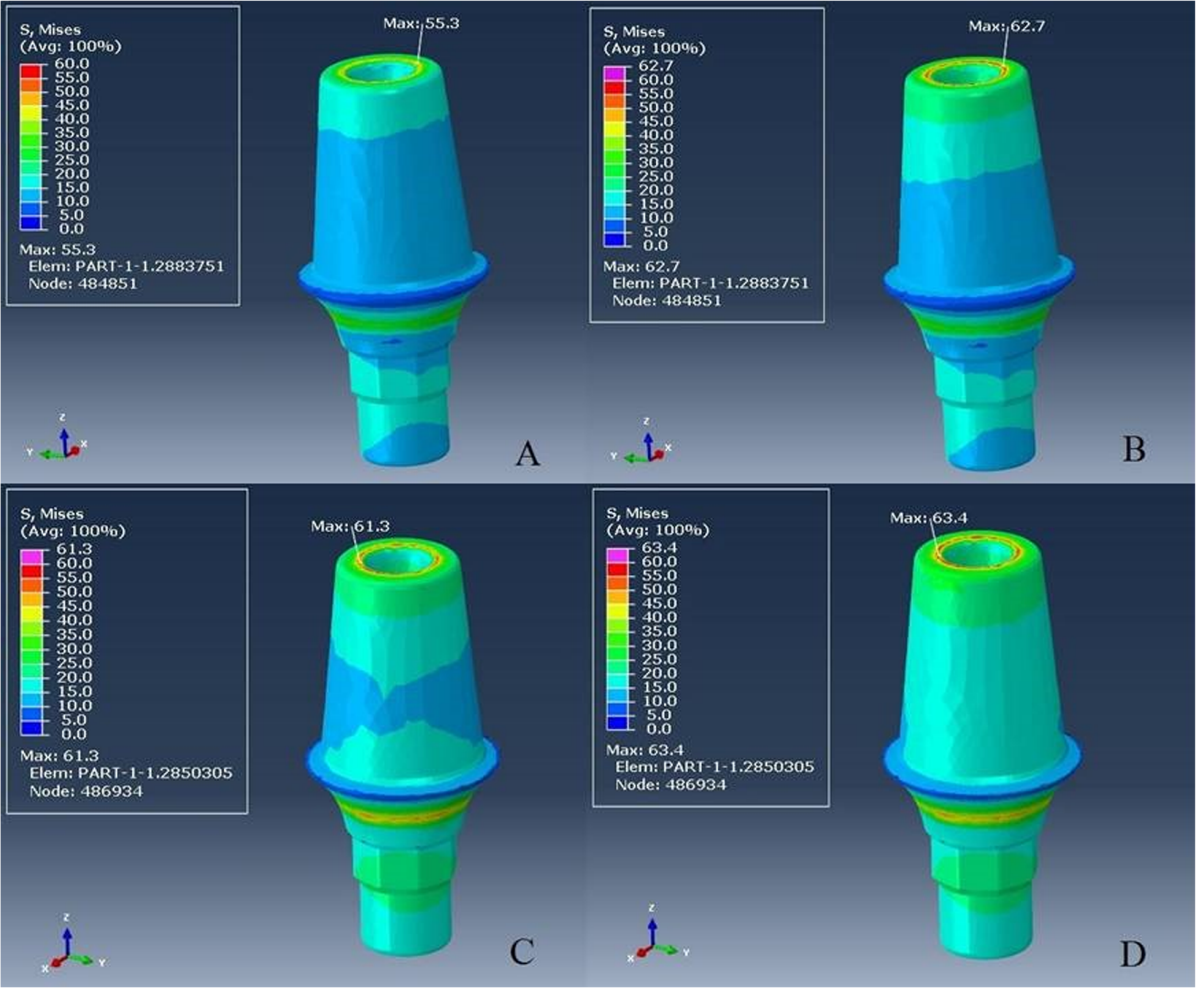
**a**–**d** Von Mises stress distribution on abutment. From **a** to **d**: L-M, ZL-M, L-V, and ZL-V respectively. Von Mises stresses were relatively similar and concentrated at the coronal part of the abutment in all groups

**Fig. 6:**
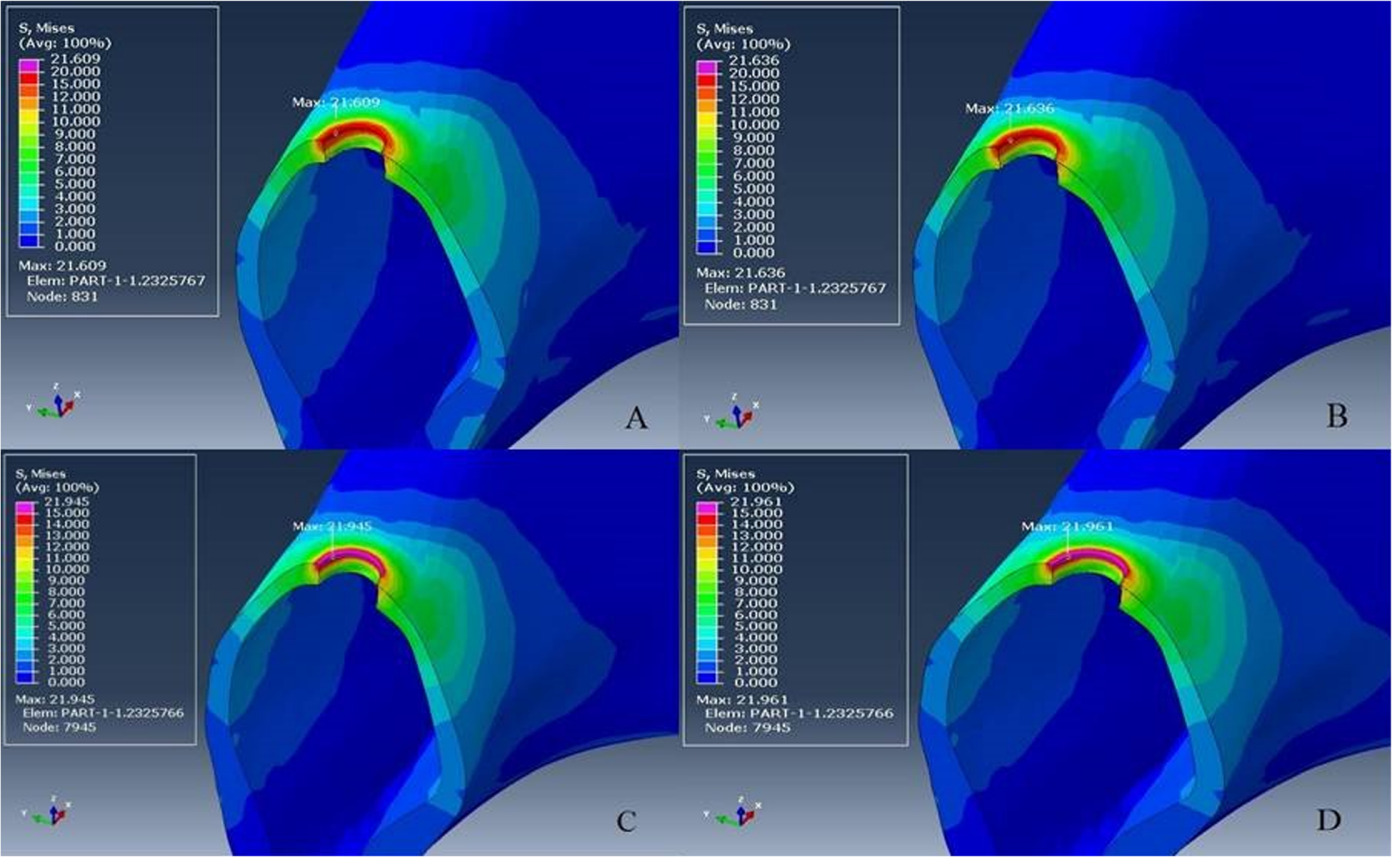
**a**–**d** Von Mises stress distribution on bone. From **a** to **d**: L-M, ZL-M, L-V, and ZL-V respectively. The stress concentration occurred in the cortical bone around the neck of the implant. Groups L-M and ZL-M were quite similar and reduced stress

Von Mises stresses were relatively similar and concentrated at the coronal part of both implants (Fig. 4a–d) and abutments in all groups (Fig. 5a–d). All stresses such as tensile, compressive, and Von Mises stresses were concentrated in the cortical bone around the implant neck (Fig. 6a–d). The results of groups L-M and ZL-M were quite similar and considerably reduced all stresses.

## Discussion

Implant-supported restorations have been accepted as an alternative treatment for the rehabilitation of edentulous spaces [22–24]. Despite the high success rates, implant failures are inevitable and classified as early or late implant failures. Late implant failures are observed after prosthetic restoration which is primarily related to biomechanical complications. Since occlusal loads are transferred to the bone interface via prosthesis, many factors have a biomechanical effect on the stress distribution on the bone-implant-prosthesis complex [25]. Among these factors, the effect of prosthetic restoration material was investigated in the present study by comparing the stress distribution and failure load of CAD/CAM-manufactured current glass ceramics.

Fracture of ceramic restorations is one of the most common problems observed in clinical use. Therefore, a ceramic crown should have sufficient fracture strength during oral function. To solve this problem, laboratory tests can be used as they facilitate the evaluation of the fracture strength of materials with crown-shaped specimens [26]. Although laboratory tests should be comparable to intraoral conditions, there may be some limitations such as dynamic load and thermal effect [27] as in our study. For this reason, these effects should be evaluated in further studies. In addition, they do not adequately simulate the clinical fractures of ceramic restorations. Cracks occur at the cervical margin for clinical crowns, but in laboratory tests, contact damage occurs with the loading device [28]. This was confirmed by our study as all fractures started from the occlusal surface.

LDS ceramic has been commonly used in wide range indications [29]. Despite the low opacity of the ceramic, it can be veneered to improve esthetic and sufficient veneer support [30]. A long-term clinical follow-up study indicated that the survival of veneered LDS restorations was 97.4% for 5 years, and the anterior and posterior regions were 93.8% and 100% for 8 years respectively [31]. In literature, it has been stated that the failure load of LDS crowns was higher than veneered zirconia [32–34] and could be comparable with metal ceramic systems [32]. Doğan et al. evaluated the fracture strength of different CAD/CAM-manufactured crowns and concluded that the monolithic LDS crowns had the highest fracture resistance [11]. Present study confirmed as monolithic LDS crowns demonstrated so satisfying failure load values. On the other hand, veneer application caused a significant decrease in the failure load of LDS crowns (L-M 2891 N; L-V 2077 N) in accordance with the study of Zhao et al. [30].

IPS e.max Ceram is a veneering ceramic compatible with both lithium disilicate and zirconia cores. Since the coefficient of thermal expansion of IPS e.max Ceram is closer to that of lithium disilicate, its use with zirconia core would probably result in some fractures [35] because the coefficient of thermal expansion differences between two materials causes the residual stresses and decreases the bond strength which can result in failures [36].

In group L-V, the only failure mode was bulk fracture instead of chipping that is observed in zirconia restorations. This finding confirmed the compatibility between e.max Ceram and e.max CAD core. In addition, crowns of the group ZL-V exhibited bulk fracture, as in monolithic crowns, consistent with previous studies [30, 32]. Zhao et al. explained that the integration of glass phases between the glass ceramic core and veneering porcelain during the firing procedure makes the multilayered structure a homogeneous material [30].

Recently, a new CAD/CAM material, zirconia-reinforced lithium silicate ceramics (ZLS), has been marketed. ZLS ceramics combine the mechanical strength of polycrystalline ceramics with the esthetic features of glass-ceramics [17]. They are milled in a precrystallization phase and can be manually veneered as in LDS ceramics [15]. Elsaka and Elnaghy indicated that ZLS ceramics revealed higher mechanical properties compared to LDS ceramics [18]. Similar results were presented in a study of Traini et al. as it was concluded that ZLS was comparable to that of existing zirconia-based ceramics and was suitable for oral function even in the posterior regions [14]. In the literature, there have been few studies on this ceramic [15, 17, 37, 38] and a limited number of them include the failure load of the material [16, 39]. In one of these studies, Preis et al. compared the fracture strength of ZLS molar crowns (adhesive cementation 2612 ± 853 N; conventional cementation 1848 and 1891 N) with LDS crowns (adhesive cementation 2528 ± 668 N) and concluded that they could be compared with LDS ceramics. Although adhesive cementation increased the fracture strength of the material, it was not statistically significant [16]. The difference from the present study was Celtra Duo (DeguDent) which has lower flexural strength than Vita Suprinity and no need for crystallization. In another study, fracture strength of different monolithic all ceramic implant-supported crowns were compared, and the highest values were observed in ZLS ceramic crowns (3056 ± 642 N; IPS e.max CAD crowns 2377 ± 572 N). It was also concluded that comparison of luting agents showed no significant difference in fracture strength [39]. On the contrary, the failure load of group ZL-M (1750.28 ± 314.96 N) was significantly lower than group L-M (2891.88 ± 410.12 N), and the first hypothesis was rejected. Different results may be associated with cementation type or restoration design (differences in abutment type, porcelain thickness, cusps size, etc.).

Within the limitation of the present study, conventional cementation was used in contrast to cementation of glass ceramic restorations in clinical use. The luting agent fills the voids between the abutment and the inner surface of the crown and eliminates any premature contacts which may cause stress concentrations [27]. Based on this, a conventional cement was used to focus on comparing materials without cement effect. Therefore, the effect of different cements should be evaluated in further studies.

Veneer application provided additional strength to the ZLS crowns in contrast to the LDS crowns. The higher failure load of the veneered ZLS crowns (2202.55 N; group L-V 2077.37 N) may be associated with the higher flexural strength of the veneering porcelain VM-11 (100 MPa; emax Ceram 90 MPa) [40]. These veneered groups had a statistically significant difference from the monolithic groups that caused the rejection of the second hypothesis. On the other hand, the third hypothesis was accepted because there was no statistically significant difference between these veneered groups.

The finite element analysis (FEA) has been widely used in implant dentistry to evaluate the effect of both biomechanical and clinical factors on implant success. This analysis identifies stresses and displacements on the prosthesis-implant-bone complex which can be unachievable for other biomechanical methods [41–43]. Therefore, in this study, FEA was used to compare the stress distribution of different glass ceramics and the effect of veneering on the prosthesis-implant-bone complex.

Since the supporting bone is affected by the magnitude and direction of transferring stress to the implants, a veneering material has a considerable effect on the stress distribution of the prosthesis-implant-bone complex because the non-uniform distribution of occlusal forces can lead to osseointegration failure [44]. However, according to our FEA results, different veneering materials did not affect the stress distribution. In order to evaluate stress concentration areas, our results have been in accordance with previous studies as stresses were concentrated at the crestal bone, implant neck level [45–47]. Although, different restorative materials demonstrated similar stress distribution on the abutment, implant, and bone, it is possible to conclude that monolithic crowns reduced the stress concentration on the peri-implant bone (Fig. 6a–d). This situation was supported by decreased stress concentration on the implant with monolithic restorations. The possible reason is that the stresses were more concentrated on the ceramic surface and reduced the load transmission to the implant and finally to the bone.

Zheng et al. compared the stress distribution of the same veneering ceramic on different cores and concluded that the zirconia core was clearly different from other materials with higher tensile stresses at the veneer core interface [48] because the increasing differences between the elasticity modulus of the core and the veneer transmitted higher stress concentrations to the cores [49]. Consistent results were observed in our study as e.max CAD, which has greater elasticity modulus difference (e.max CAD-e.max Ceram 95–64 GPa; Vita Suprinity-VM-11 65–65 GPa), showed higher stress concentration (33.8 MPa) than Vita Suprinity core (30.7 MPa).

In order to validate the model, it is possible to compare the FEA results with the in vitro test results and previous studies in the literature [50]. FEA showed similarity with the in vitro test in the stress type. Compressive stress values varying from − 1074 to − 1110 MPa, higher than tensile stresses (322–374 MPa), for crown models and compression loads (negative values) were predominant as in the load-to-failure test. The stresses were concentrated in the central groove and fissure as observed in the fracture pattern on the remnants of the load-to-failure test. After the load-to-failure-test, there was no chipping or fracture in the cervical area. This was confirmed by FEA as no stress was concentrated in this area (Fig. 3a–d). Additionally, veneered e.max CAD crowns with higher stress concentration on the cores had a lower failure load than veneered Vita Suprinity crowns. Similar to the FEA results of the previous studies, different restorative materials did not change stress distributions on the bone [44, 49] and they were concentrated in the cortical bone around the implant neck in accordance with the literature [21, 46, 47]. The relation between these findings validates our FEA model.

## Conclusions

Within the limitation of the present study, it can be concluded that the restoration design affected the failure load of ceramics. Monolithic design had a statistically significant effect on the failure load of two different ceramics (LDS > ZLS). Veneer application had opposite effects on two different ceramics which increased the failure load of ZLS and reduced it for LDS without a statistically significant difference. Nevertheless, both materials are suitable for implant-supported crown as the failure loads of whole crown restorations were higher than posterior occlusal loads. Different restorative materials did not influence the stress distribution, but monolithic restorations reduced the stress concentration on the implant and bone.

## Data Availability

All data generated or analyzed during this study are included in this published article.

## Abbreviations

CAD/CAM: Computer-aided design/computer-aided manufacturing
LDS: Lithium disilicate
ZLS: Zirconia-reinforced lithium silicate
FEA: Finite element analysis
Pmax: Tensile strength
Pmin: Compressive strength
